# Nephrotic Syndrome in Adult Patients With COVID-19 Infection or Post COVID-19 Vaccine: A Systematic Review

**DOI:** 10.7759/cureus.29613

**Published:** 2022-09-26

**Authors:** Ivan Cancarevic, Mahmoud Nassar, Luis Medina, Angelica Sanchez, Avish Parikh, Asma Hosna, Bhavana Devanabanda, Mallorie Vest, Fatima Ayotunde, Muhammad Ghallab, Ismail Omran

**Affiliations:** 1 Internal Medicine, Icahn School of Medicine at Mount Sinai/NYC Health+Hospitals/Queens, New York, USA; 2 Internal Medicine, Queens Hospital Center, New York, USA; 3 Internal Medicine, Universidad Autónoma de Santo Domingo, San Francisco de Macorís, DOM; 4 Internal Medicine, Mount Sinai Hospital, New York , USA; 5 Integrative Medicine, Icahn School of Medicine at Mount Sinai/NYC Health+Hospitals/Queens, New York, USA

**Keywords:** covid 19, coronavirus vaccine, primary membranous nephropathy, minimal change, focal segmental glomerulosclerosis (fsgs), coronavirus disease 2019, nephrotic syndrome

## Abstract

Nephrotic syndrome is a condition characterized by damage to podocytes that results in significant proteinuria, edema, hyperlipidemia, and hypercoagulability. Infections and malignancies are frequently associated with nephrotic syndrome. The COVID-19 virus has been associated with several atypical presentations of upper respiratory infections and acute kidney injury. Considering that COVID-19 causes systemic inflammatory changes, it seems plausible that it may also lead to nephrotic syndrome. This study aimed to investigate if an association between COVID-19 and the different types of nephrotic syndromes exists. Data were extracted into a spreadsheet. Statistical analysis was performed using Statistical Package for Social Sciences (SPSS, IBM Corp., Armonk, NY, USA).

We performed a systematic search of PubMed/Medline and Embase databases using both medical subject headings (MeSH) and regular keywords associated with COVID-19 and nephrotic syndrome, including different types of nephrotic syndromes. The search was performed on 17th December 2021. We included case reports and case series about adult patients who developed findings suggestive of nephrotic syndrome shortly after infection or vaccination. We excluded cases involving children, pregnant women, articles written in languages other than English, and those that were not retrievable. The relevance and quality of identified articles were assessed.

We included 32 articles in the study, primarily case reports and case series. In our study, COVID-19 and the COVID-19 vaccine have been associated with the development of nephrotic syndrome, primarily a collapsing form of focal segmental glomerulosclerosis, although other forms have been observed as well. There was little consistency in patient histories, clinical presentations, clinical courses, or treatment regimens, although it appeared that most cases eventually resolved. More cases need to be reported and analyzed before more definitive conclusions can be reached.

In conclusion, nephrotic syndrome is a possible complication of both COVID-19 infection and the COVD-19 vaccine and should be considered in patients exhibiting sudden onset edemas or deterioration in kidney function. While the majority of cases respond to standard treatment, clearer guidelines will need to be developed once more data is available.

## Introduction and background

An outbreak of pneumonia of unknown etiology was discovered in December 2019 in Wuhan, China. Samples were obtained from affected patients, and molecular analysis showed a new coronavirus, initially named 2019-nCoV, subsequently renamed by the World Health Organization (WHO) as COVID-19. This new coronavirus strain is the seventh member of the coronaviridae family that can infect humans. It is a highly pathogenic and contagious virus, which prompted the WHO to declare it a public emergency of international concern on January 30th, 2020, and a pandemic by March 11th, 2020 [1,2]. Furthermore, by August 8th, 2022, there have been 591,407,978 confirmed cases of COVID-19 worldwide, with 6,442,204 reported deaths [3]. Although several transmission routes are known, respiratory droplets and close contact are the leading modes of transmission [4]. The most common presenting symptoms are fever, loss of smell, cough, and fatigue, with a small percentage of patients presenting with severe organ damage and/or death [5,6].

Since the beginning of the pandemic, several measures to decrease the spread of the virus have been implemented, including social distancing, the use of masks, and regional lockdowns, among others. In addition, several treatments were studied, such as antivirals drugs (i.e., lopinavir, ritonavir, and remdesivir) which showed some efficacy against the virus, and immunomodulators (i.e., tocilizumab, eculizumab, dexamethasone, colchicine) which appeared to reduce the hyperinflammatory state secondary to the cytokine storm caused by this viral illness [5-8]. However, as there is no medication that directly kills the virus, vaccines became the last hope for stopping the pandemic [9]. As a result, the Food and Drug Administration (FDA) issued an emergency use authorization for Pfizer-BioNTech (Cominarty/BNT162b2) vaccine on December 11th, 2020; for Moderna (mRNA-1273) vaccine on December 18th, 2020; and for Johnson & Johnson's Janssen vaccine (JNJ-78436735) on February 27th, 2021 [10].

Pfizer-BioNTech and Moderna, which have an effectiveness of 95% and 94.1%, respectively, are messenger ribonucleic acid (mRNA) vaccines that trigger an immune response against the spike protein of COVID-19. The vaccines are lipid nanoparticle-encapsulated mRNA which enters the cell via endocytosis. Once in the cytoplasm, the mRNA escapes the endosome and the lipid nanoparticle that protects the mRNA from degradation and is translated by ribosomes into spike protein. Later, the spike protein follows one of two pathways: 1) it is partially degraded in proteasomes to peptides, then loaded on major histocompatibility complex (MHC) class I for interaction with immune cells; or 2) it is secreted into the extracellular space, internalized into antigen-presenting cells via endocytosis, displayed in MHC class II and presented to immune cells which start to produce antibodies against COVID-19 [9,11-13]. The Johnson & Johnson's Janssen vaccine is a viral vector vaccine with an effectiveness of 66.3% that uses a replication-deficient engineered virus to express the genetic sequence of the antigen of interest into the host cells [14].

The most common side effects of the COVID-19 vaccines include pain, swelling, and redness in the arm where the vaccine is injected, as well as tiredness, muscle pain, headache, fever, chills, and nausea. In addition, less common severe side effects are starting to be recognized, such as nephrotic syndrome, a clinical entity characterized by proteinuria, hypoalbuminemia, edema, and hyperlipidemia, among other complications [12,13,15,16]. This systematic review presents several cases of nephrotic syndrome that presented post-COVID-19 infection or vaccination. Although the exact mechanism of nephrotic syndrome in these settings has not yet been elucidated, some hypotheses have surged. Microscopic evaluation of kidney specimens in some patients has shown spherical particles that resemble the virus in the cytoplasm of podocytes which suggests a direct viral cytopathic effect, similar to HIV-associated glomerulopathy [17]. Another hypothesis is that angiotensin-converting enzyme 2 receptors (which help COVID-19 invasion to target cells) present in podocytes and proximal convoluted tubules could explain the propensity of these cells to be affected [17,18]. Therefore, clinicians need to consider the possible association between nephrotic syndrome and COVID-19 infection or vaccination so that they can be vigilant to the presence of signs and be prepared to give the appropriate management.

## Review

Methods

This article follows the Preferred Reporting Items for Systematic Reviews and Meta-Analyses (PRISMA) guidelines for systematic reviews. A systematic literature search was conducted using the PubMed/Medline and Embase databases. The search was conducted from inception to 17th December 2021. The following search strategy and combinations of medical subject headings (MeSH) terms and regular keywords were used: (“COVID-19"[Mesh] OR "SARS-CoV-2"[Mesh] OR "COVID-19 Vaccines" [Mesh] OR "BNT162 Vaccine" [Mesh] OR "2019-nCoV Vaccine mRNA-1273" [Mesh] OR "Ad26COVS1"[Mesh] OR "COVID19" OR "COVID" OR "coronavirus 2019" OR "novel coronavirus" OR "SARS-CoV-2" OR "new coronavirus" OR "COVID2019" OR "COVIDvaccines" OR "mRNA vaccines" OR "coronavirus vaccines" OR "SARS-CoV-2 vaccines" OR "Pfizer vaccine" OR "Pfizer-BioNTech vaccine" OR "Moderna vaccine" OR "Janssen vaccine" OR "Johnson and Johnson vaccine") AND ("Nephrotic Syndrome" [Mesh] OR "Glomerulosclerosis, Focal Segmental" [Mesh] OR "Nephrosis, Lipoid" [Mesh] OR "Glomerulonephritis, Membranous" [Mesh] OR "nephrotic syndrome" OR "focal segmental glomerulosclerosis" OR "focal sclerosing glomerulonephritis" OR "FSGS" OR "minimal change disease" OR "membranous nephropathy" OR "Heymann nephritis").

The results were exported into the Covidence platform, which removed duplicates automatically. Two independent reviewers assessed articles in two steps, first by reviewing titles and abstracts only, followed by full-text analysis. The conflicts were resolved through direct communication between reviewers or by a third reviewer's vote. We excluded all cases of pre-existing nephrotic syndrome and cases involving children or pregnant women. Case series were included if relapses constituted the minority of cases. We also excluded articles written in languages other than English. We included case reports, case series, cross-sectional studies, prospective and retrospective case-control, and cohort studies. Reviews and meta-analyses were excluded. Quality assessment was performed using the Newcastle-Ottawa scale for case-control and cohort studies and the 'Checklist for Case Reports - Joanna Briggs Institute' for case reports and case series. We included studies that scored at least two points below the maximum on either scale. Abstracts were included if they met the same quality requirements as manuscripts. A spreadsheet was used to extract the data, and relevant features of the patients' presentations, laboratory values, and disease courses were recorded as reported in the original study. Where possible, laboratory values were converted into US units. The data were then analyzed and reported descriptively. The mean and standard deviations were calculated for numeric variables using SPSS. The findings were presented in tables that were generated. No assumptions were made, and any data that was not directly reported was recorded as not applicable (NA). Each analysis was conducted only among the studies in which the variable of interest was reported.

Results

**Figure 1:**
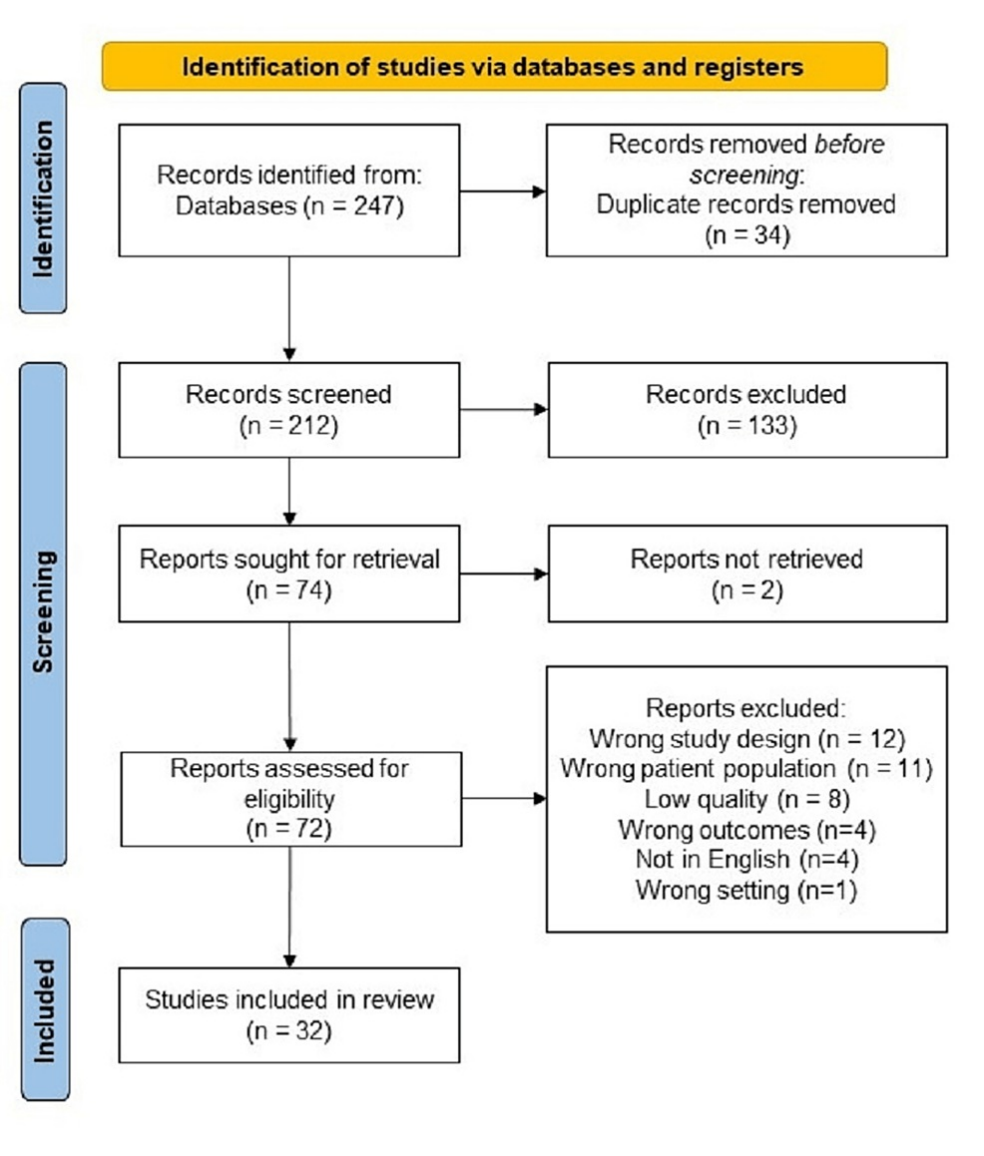
PRISMA flow diagram of literature research PRISMA: Preferred Reporting Items for Systematic Reviews and Meta-Analyses

A total of 247 studies were imported from Medline/PubMed and Embase databases, and 34 duplicates were removed, leaving 212 studies for screening. Two independent reviewers found 133 of these studies irrelevant, while 74 were screened for full text. Out of those, 32 were included in the final analysis (Figure 1) [15,17-46]. We removed 12 studies because the study designs were incorrect, 11 because they were related to the wrong patient population, and eight because they did not meet the quality assessment requirements. Further reasons included different outcomes than the ones we are studying, languages other than English, a reference to the pediatric population, and no follow-up information. No obvious bias was detected. We extracted the data in a spreadsheet and collected demographic information (age, gender, ethnicity), pertinent medical history (history of chronic kidney disease (CKD), malignancies, chronic infections, use of non-steroidal anti-inflammatory medications, kidney transplantation), relevant laboratory data on presentation and at baseline (serum creatinine, serum albumin, urine protein), the disease course, including the number of hemodialysis sessions, the number of intensive care unit (ICU) admissions, and the number of relevant treatments prescribed. The follow-up data included the follow-up period, the need for hemodialysis, serum creatinine, and urine protein at discharge. All units were converted to those typically used in the United States. For some variables, conversion was impossible, and they were recorded as reported in the original study.

A total of 76 patients were included in this study, 56 (73.3%) male and 20 (26.3%) female. The mean age of the sample is 52.7±16.4, ranging from 19 to 83 (Table 1).

**Table 1:** Quantitative analysis of included studies N: Total number of patients, SD: Standard deviation

	N	Range	Minimum	Maximum	Mean±SD
Age (in years)	76	64	19	83	52.74±16.46
Days till onset	38	119	1	120	9.71±19.38
Serum albumin	66	4	1	5	2.18±0.96
Baseline creatinine	55	2.57	0.6	3.17	1.26±0.52
Creatinine	70	19.4	0.6	20	5.13±4.41
Follow-up time	61	239	1	240	43.21±38.88
Creatinine at last follow-up	41	15.9	0.7	16.6	2.95±3.23

The most common races among the patients are as follows: 35 (46.1%) African American patients and 19 (25%) Caucasian patients. A total of 50 (65.8%) patients had confirmed COVID-19 infection, and 46 (92%) were symptomatic. Twenty-six (34.2%) patients had received the COVID-19 vaccine. The symptoms began after the first dose of the vaccine in 15 (19.7%) individuals and after the second dose in 10 (13.2%) individuals.

Five patients (6.6%) had a history of kidney transplantation. Chronic infection was reported in six (7.9%) cases (hepatitis C virus (HCV), human immunodeficiency virus (HIV), hepatitis B virus (HBV), malaria, schistosomiasis, and streptococcal infection). There were 56 (73.3%) patients reported as not having CKD, 12 (15.7%) cases reported to have CKD, and eight (10.5%) cases reported to have end-stage renal disease (ESRD). Three patients had prostate cancer. There were two cases of cervical cancer and one case of cutaneous mycosis fungoides. Non-steroidal anti-inflammatory drugs (NSAID) use was reported in only one case report.

Among 76 reported cases, only in 38 was the interval between infection or vaccination and the onset of nephrotic syndrome reported. The mean interval is 9.71±19.37 days. This ranges from one day to four months. Only one patient required intensive care admission. At the time of presentation, 36 patients had acute kidney injury (AKI). Twenty-seven (35.5%) of the patients presented with edema. Both AKI and proteinuria were observed in 15 (19.7%) patients. Three patients presented with gross hematuria. Two patients presented with anuria/oliguria and one with anasarca (Table 2).

**Table 2:** First nephrotic symptoms AKI: Acute kidney injury, HTN: Hypertension, NA: Not available

First nephrotic symptoms	Frequency (%)
Abdominal pain, nausea, anorexia	1 (1.3%)
Abdominal pain, vomiting, AKI	1 (1.3%)
AKI	13 (17.1%)
AKI, hematuria, proteinuria	1 (1.3%)
AKI, proteinuria	13 (17.1%)
AKI, skin rash	1 (1.3%)
Anasarca	1 (1.3%)
Anuria	1 (1.3%)
Edema	20 (26.3%)
Edema, AKI	5 (6.6%)
Edema, weight gain	1 (1.3%)
Elevated creatinine	1 (1.3%)
Gross hematuria	3 (3.9%)
HTN	1 (1.3%)
HTN, edema	1 (1.3%)
Hypoxia	1 (1.3%)
Joint pain, proteinuria	1 (1.3%)
NA	6 (7.9%)
Oliguria	1 (1.3%)
Proteinuria	1 (1.3%)
Proteinuria, AKI	2 (2.6%)
Total	76 (100%)

The mean serum albumin level in the pool of patients was 2.18±0.96 g/dl. The mean baseline creatinine concentration for the patients included in this study is 1.25±0.51 mg/dL. The mean serum creatinine level is 5.13±4.41 mg/dL. A renal biopsy revealed that two patients had anti-glomerular basement membrane (GBM), and 24 patients had collapsing focal segmental glomerulosclerosis (FSGS). Thirteen patients had minimal change disease (MCD). Nine patients had membranous nephropathy. Five patients had immunoglobulin A (IgA) nephropathy (Table 3). The treatment of COVID-19 included tocilizumab for six patients, hydroxychloroquine for seven patients, convalescent plasma for one patient, and steroids for nine patients. 

**Table 3:** Biopsy Findings GBM: Glomerular basement membrane, ATI: Acute tubular injury, FSGS: Focal segmental glomerulosclerosis, ATN: Acute tubular necrosis, IgAN: IgA Nephropathy, AIN: Acute interstitial nephritis, MCD: Minimal change disease, MPO-ANCA: Myeloperoxidase anti-neutrophil cytoplasmic antibodies

Biopsy Findings	Frequency (%)
Anti-GBM	2 (2.6%)
ATI	5 (6.6%)
Collapsing FSGS	14 (18.4%)
Collapsing FSGS, ATN	9 (11.8%)
Collapsing FSGS, membranous nephropathy, ATN	1 (1.3%)
Diabetic glomerulosclerosis, ATN	3 (3.9%)
FSGS	7 (9.2%)
Glomerular sclerosis	1 (1.3%)
Henoch-Schoenlein purpura nephritis, ATN	1 (1.3%)
IgAN	4 (5.3%)
IgAN, AIN	1 (1.3%)
Infarction	1 (1.3%)
Lupus nephritis	1 (1.3%)
MCD	13 (17.1%)
Membranous nephropathy	7 (9.2%)
Membranous nephropathy, ATN	1 (1.3%)
MPO-ANCA	1 (1.3%)
Not done	1 (1.3%)
Podocyte effacement	2 (2.6%)
T-cell mediated rejection	1 (1.3%)
Total	76 (100%)

For the treatment of nephrotic syndrome, nine patients were given angiotensin-converting enzyme (ACE) inhibitors, eight patients were given diuretics, and five patients underwent hemodialysis. Thirty-eight (51.3%) patients received corticosteroids, while 39 (51.3%) did not. Twenty-four (18.24%) patients required hemodialysis shortly after diagnosis (Table 4). The average follow-up period is 43.21±38.8 days. Regarding prognosis, six patients experienced complete resolution; in 15 patients, their renal function worsened, eight remained stable, 33 improved, and one died. At follow-up, only 16 patients (21.1%) required dialysis. The mean creatinine level at follow-up was 2.95±3.23 mg/dL.

**Table 4:** COVID-19 treatment IVIG: Intravenous immune globulin, NA: Not available

COVID-19 Treatment	Frequency (%)
Azithromycin, ceftriaxone	1 (1.3%)
Azithromycin, hydroxychloroquine	1 (1.3%)
Convalescent plasma	1 (1.3%)
Hydroxychloroquine	2 (2.6%)
Hydroxychloroquine	2 (2.6%)
IVIG	1 (1.3%)
Lenzilumab, steroids	1 (1.3%)
None	45 (59.2%)
NA	6 (7.9%)
Plasmapheresis, steroids	1 (1.3%)
Prednisone	1 (1.3%)
Solumedrol, mechanic	1 (1.3%)
Steroids	5 (6.6%)
Steroids, hydroxychloroquine	2 (2.6%)
Tocilizumab	1 (1.3%)
Tocilizumab, hydroxychloroquine	3 (3.9%)
Tocilizumab, IVIG, steroid	1 (1.3%)
Tocilizumab, steroid	1 (1.3%)
Total	76 (100%)

Discussion

Patient Characteristics

Among the studies we analyzed, nephrotic syndrome has been reported both following COVID-19 infection and vaccination. However, there were more cases reported following the infection than following the vaccine. Of the 78 patients analyzed, 26 developed nephrotic syndrome after receiving the vaccine, while the rest developed it following infection with COVID-19. Those findings are in line with what is currently known about other immune-mediated complications of COVID-19 infection and vaccines, such as myocarditis, which still occurs much more frequently following the infection than following the vaccine [47].

The age of onset of nephrotic syndrome, in general, varies, with different types being more prevalent in different age groups. For instance, while MCD is common in childhood, FSGS and membranous nephropathy generally occur in middle-aged and older adults [48]. Most of the patients in our review were in their 40s and 50s at the time of the diagnosis. Patients between 40 and 60 years of age accounted for almost half of all the cases reported in studies included in this review. However, this result might be confounded by the fact that younger individuals would be more likely to recover from COVID-19 without ever having tested positive due to the scarcity of specific symptoms, and their new-onset nephrotic syndromes may not be associated with COVID-19 [49]. Furthermore, focal segmental (collapsing) glomerulopathy was the most common type of nephrotic syndrome reported, and it is generally less common in younger adults. Slightly more cases were reported among African-Americans compared to other races, but that could again be explained by the general difference in the incidence of FSGS. Overall, only 20 of the cases were reported among females, while the rest occurred in males. It should, however, be noted that several larger studies, such as Nasr et al. reported the vast majority of their cases in males [41]. Despite the presence of nephrotic syndrome in renal transplant recipients, there was no difference in mortality outcomes [49-51]. 

Overall, we can conclude that patient characteristics among the reported cases of nephrotic syndrome following COVID-19 infection or vaccination match what would be expected in any population of patients who develop nephrotic syndrome. However, it should be noted that our study excluded pediatric patients even though nephrotic syndrome, namely MCD, does occur in this population. Children were also the last population to be offered the vaccine against COVID-19. It should also be noted that, while the period in which the COVID-19 vaccine has been available only constitutes about half the time COVID-19 has been present, the number of doses of COVID-19 in the countries that the studies originate in significantly surpasses the number of COVID-19 cases. Therefore, it does appear to be the case that the association between the infection and the development of nephrotic syndrome is stronger than the association between the vaccination and the development of the nephrotic syndrome.

Medical Histories

It is known that nephrotic syndromes are frequently associated with other co-morbidities, especially of infectious and malignant etiology. However, among the patients reported to have developed nephrotic syndrome after either a COVID-19 infection or vaccination, we could not find a clear link between chronic infections or a history of cancer. Only six of the patients had recorded histories of chronic infections, and no two patients had the same type of chronic infection. Malhotra et al. reported a case of FSGS in a patient with a known history of HIV, a known risk factor for FSGS [27]. The HIV infection can be regarded as a possible confounder since this patient would have been at increased risk regardless of the COVID-19 infection. If the association between COVID-19 and FSGS does exist, however, it is plausible that patients with HIV who develop COVID-19 would be at a particularly high risk of FSGS, but that claim would require significantly more data to prove. In addition, histories of both chronic hepatitis B and C were found among studied patients who developed FSGS, but those infections are less commonly associated with FSGS [34,46].

Five patients had a history of cancer, and the only cancer appearing more than once was prostate cancer; however, all three patients were above the age of 60, and at that age, a large number of men developed slow-progressing types of prostate cancer at that age [41,46]. Only one patient had a reported history of NSAID use [41]. It should be noted that the details of the medical histories of many of the patients may not have been sufficiently detailed to describe such histories even if they existed. Moreover, NSAIDs can be purchased over the counter. Many of the cases were reported among patients without prior history of CKD. However, some patients with elevated creatinine at baseline were not recorded as patients with a history of CKD. Most occurred in native kidneys, although there were five reports of nephrotic syndrome in transplanted kidneys. The actual relevance of this finding is questionable because the number of people with transplanted kidneys in the population is relatively low, so even 5/78 appears like a disproportionately large number. Case reports of patients with prior nephrotic syndrome were excluded. However, this criterion was not applied in all the case series that were included, and so it is possible that some of the patients had relapses.

In summary, from the data that has been reported in the literature so far, it is difficult to establish whether any particular aspect of one's medical history predisposes them to a nephrotic syndrome in the setting of COVID-19 infection or vaccination. Insufficient detail in reporting past medical histories affected the findings, and more detailed case reports and case series would be needed in the future to paint a clearer picture of any possible associations. The feasibility of a case-control study to assess the odds ratio for any of the aforementioned variables is questionable due to an incomplete understanding of the actual prevalence of the condition.

Clinical Findings and Management

Nephrotic syndrome is characterized by protein loss in the urine leading to characteristic findings of widespread edemas, hypercoagulability, hyperlipidemia, and other metabolic disruptions. Typical symptoms of COVID-19 are upper and lower respiratory symptoms, but it frequently affects other organs. Any disease that causes immunosuppression makes an individual more prone to severe infections, including COVID-19. Therefore, it is important to establish whether nephrotic syndrome occurred before or after the onset of COVID-19, and we excluded cases where there is a strong suspicion that nephrotic syndrome was present prior to the onset of COVID-19-related symptoms. Among the reported cases, most patients developed nephrotic syndrome within seven days; however, this is a difficult number to interpret as there is not a well-defined point where people seek medical care or get tested for COVID-19, and while some may present early, others may not, increasing the risk of bias. Moreover, many cases were reported in the first three days. A possibly more accurate assessment would be to base it on the day of the onset of symptoms, but in that case, the findings would depend on subjective symptom recollection, raising the possibility of recall bias. The longest gap between the two diagnoses that was reported was four months [52]. Another possible issue complicating the interpretation of data, in this case, is the question of the gap between when the pathophysiological process eventually leads to nephrotic syndrome starts developing and the time when clinical or biochemical findings can be detected. It could be the case that, in some cases, the initial trigger for the nephrotic syndrome occurred before the onset of COVID-19. Such findings could make cases with a wider gap between COVID-19 or vaccination and nephrotic syndrome development more relevant.

The most common initial symptom of nephrotic syndrome was the onset of edema; however, several patients were only identified when they were found to be proteinuric despite no complaints. Proteinuria was often severe, with a mean value of approximately 12 grams per 24 hours. Additionally, some patients presented with elevated serum creatinine levels and AKI as their initial findings. It should be noted that in some of the cases, patients were found to have findings consistent with a form of nephrotic syndrome after biopsies, but they never developed nephrotic-range proteinuria. The severity of COVID-19 infection varied widely among the cases studied, and there was no clear association between nephrotic syndrome and any particular severity of COVID-19 infection. One of the patients died of the infection, which correlates with the assumed mortality rate of COVID-19 of between 1% and 2% [41]. Likewise, ICU admission was not overrepresented among the cases compared to what would be expected in any general population of COVID-19 patients of the same age. A wide range of pathological findings was reported, although FSGS was the most commonly reported finding, especially the collapsing type, the regular type was also reported. Thirty-one of 78 patients were reported to have forms of FSGS. Minimal change disease and membranous nephropathy (mostly phospholipase A2 receptor (PLA2R) antibody (Ab)-associated "primary type") were also reported multiple times. Focal segmental glomerulosclerosis is also the most common cause of primary nephrotic syndrome in the general population, which should be considered when interpreting these findings.

The conservative treatment for nephrotic syndrome consists of renin-angiotensin-aldosterone system (RAAS) inhibition and diuretics. Steroids are prescribed for more aggressive treatment. However, corticosteroids are also used to treat patients with COVID-19 pneumonia. While methylprednisolone and prednisone are typically used for nephrotic syndrome, dexamethasone is predominantly used in cases of COVID-19. The most frequently reported corticosteroid among patients with both conditions was prednisone, although the other two were also reported. Authors have reported the administration of steroids in many cases without specifying the type of steroids. No clear association could be drawn between COVID-19-specific treatments and the likelihood of renal recovery in this group of patients, although inadequate reporting could be playing a role. Other immunosuppressive medications were sometimes used as well. Historically, COVID-19 has been treated with some of these agents (e.g., hydroxychloroquine). Many patients experienced a complete or partial recovery in renal function. Notably, those who developed nephrotic syndrome after the infection appeared to be more likely to suffer residual renal damage, including, in rare instances, requiring hemodialysis at the end of the follow-up period. There were 16 reports of this, and all 16 occurred following an infection. Importantly, in 62 of the cases, the eventual kidney function was described either verbally or by showing a change in serum creatinine. In 23 of those cases, kidney function remained stable or worsened, while in the rest, it improved.

In summary, the clinical presentation and prognosis of patients with nephrotic syndrome in the setting of recent COVID-19 infection or vaccination vary widely, and almost any type of nephrotic syndrome can be precipitated by them. The most common form appears to be focal segmental glomerulosclerosis collapsing, but FSGS is also one of the most common causes of nephrotic syndrome in general adults. Moreover, the timing of onset and clinical presentation of the nephrotic syndrome was not uniform. Steroids are frequently prescribed, and it appears that most clinicians favor prednisone, the corticosteroid of choice for nephrotic syndrome, over dexamethasone, the steroid of choice for COVID-19 when both diseases occur simultaneously. Focal segmental glomerulosclerosis normally has a relatively poor prognosis, so it may be true that cases associated with COVID-19 or the vaccine are milder since, in the majority of reported cases, patients experienced partial or complete recovery of their baseline kidney function.

Limitations of the Study

This study has several notable limitations. Firstly, the majority of articles are either case reports or abstracts. Despite both being subject to the quality assessment process, there is a limited amount of detail, particularly regarding patient histories. Moreover, the overall amount of data on the topic is scarce, and the risk of publication bias exists. Due to the relative randomness in which information is obtained from each study, most of the findings can only be reported descriptively, which increases the risk of selection bias.

Future Directions

The scarcity of data within this type of study presents one of the major challenges. More cases are necessary to better understand the incidence and factors associated with the development of nephrotic syndrome in patients with COVID-19 or after COVID-19 vaccination. Additionally, more information is required to determine whether nephrotic syndrome can also be linked to other medical conditions or medications. No clear guidelines are available regarding the most appropriate treatment. Therefore, a randomized clinical trial comparing prednisone and dexamethasone in the setting of concurrent COVID-19 and nephrotic syndrome could be beneficial. A multi-center study is likely to be necessary due to the rarity of such a presentation.

## Conclusions

Both COVID-19 infection and COVID-19 vaccination can be associated with nephrotic syndrome. It most commonly presents as collapsing FSGS with significant proteinuria and edema, but other types of nephrotic syndrome and other clinical presentations have been reported. There is no clear association between any aspect of past medical histories and the development of nephrotic syndrome, nor was the severity of COVID-19 associated with the condition. When patients develop edema, rising creatinine levels, or significant proteinuria after infection or vaccination with COVID-19, the nephrotic syndrome should be considered, and treatment initiated as soon as possible since the prognosis in individuals who receive timely treatment appears to be favorable. Treatment with steroids, primarily prednisone, has usually improved or resolved symptoms. Due to the lack of uniformity in the reporting of data in the case reports analyzed, more detailed case reports and case series are needed before definitive conclusions can be made about possible associations.
